# Glycogen synthase activity in *Candida albicans* is partly controlled by the functional ortholog of *Saccharomyces cerevisiae* Gac1p

**DOI:** 10.1128/msphere.00575-24

**Published:** 2024-09-24

**Authors:** Jian Miao, David L. Williams, Michael D. Kruppa, Brian M. Peters

**Affiliations:** 1 Pharmaceutical Sciences Program, College of Graduate Health Sciences, University of Tennessee Health Science Center, Memphis, Tennessee, USA; 2 Department of Surgery, Quillen College of Medicine, East Tennessee State University, Johnson City, Tennessee, USA; 3 Center of Excellence in Inflammation, Infectious Disease, and Immunity, East Tennessee State University, Johnson City, Tennessee, USA; 4 Department of Biomedical Sciences, Quillen College of Medicine, East Tennessee State University, Johnson City, Tennessee, USA; 5 Department of Clinical Pharmacy and Translational Science, College of Pharmacy, University of Tennessee Health Science Center, Memphis, Tennessee, USA; 6 Department of Microbiology, Immunology, and Biochemistry, College of Medicine, University of Tennessee Health Science Center, Memphis, Tennessee, USA; University of Michigan Michigan Medicine, Ann Arbor, Michigan, USA

**Keywords:** *Candida albicans*, glycogen synthase, yeast, glycogen synthesis, Gac1

## Abstract

To adapt to various host microenvironments, the human fungal pathogen *Candida albicans* possesses the capacity to accumulate and store glycogen as an internal carbohydrate source. In the model yeast *Saccharomyces cerevisiae*, *Sc*Glc7p and *Sc*Gac1p are the serine/threonine type 1 protein phosphatase catalytic and regulatory subunits that control glycogen synthesis by altering the phosphorylation state of the glycogen synthase Gsy2p. Despite recent delineation of the glycogen synthesis pathway in *C. albicans*, the molecular events driving synthase activation are currently undefined. In this study, using a combination of microbiologic and genetic techniques, we determined that the protein encoded by uncharacterized gene *C1_01140C*, and not the currently annotated *C. albicans* Gac1p, is the major regulatory subunit involved in glycogen synthesis. C1_01140Cp contains a conserved GVNK motif observed across multiple starch/glycogen-binding proteins in various species, and alanine substitution of each residue in this motif significantly impaired glycogen accumulation in *C. albicans*. Fluorescent protein tagging and microscopy indicated that C1_01140Cp-GFPy colocalized with *Ca*Glc7p-tdTomato and *Ca*Gsy1p-tdTomato accordingly. Co-immunoprecipitation assays further confirmed that C1_01140Cp associates with *Ca*Glc7p and *Ca*Gsy1p during glycogen synthesis. Lastly, *c1_01140c*Δ/Δ exhibited colonization defects in a murine model of vulvovaginal candidiasis. Collectively, our data indicate that uncharacterized C1_01140Cp is the functional ortholog of the PPP1R subunit *Sc*Gac1p in *C. albicans*.

**IMPORTANCE:**

The capacity to synthesize glycogen offers microbes metabolic flexibility, including the fungal pathogen *Candida albicans*. In *Saccharomyces cerevisiae*, dephosphorylation of glycogen synthase by the *Sc*Glc7p-containing phosphatase is a critical rate-limiting step in glycogen synthesis. Subunits, including *Sc*Gac1p, target *Sc*Glc7p to α-1,4-glucosyl primers for efficient *Sc*Gsy2p synthase activation. However, this process in *C. albicans* had not been delineated. Here, we show that the *C. albicans* genome encodes for two homologous phosphatase-binding subunits, annotated *Ca*Gac1p and uncharacterized C1_01140Cp, both containing a GVNK motif required for polysaccharide affinity. Surprisingly, loss of *Ca*Gac1p only moderately reduced glycogen accumulation, whereas loss of C1_01140Cp ablated it. Fluorescence microscopy and co-immunoprecipitation approaches revealed that C1_01140Cp associates with *Ca*Glc7p and *Ca*Gsy1p during glycogen synthesis. Moreover, C1_01140Cp contributed to fungal fitness at the vaginal mucosa during murine vaginitis. Therefore, this work demonstrates that glycogen synthase regulation is conserved in *C. albicans* and C1_01140Cp is the functional ortholog of *Sc*Gac1p.

## INTRODUCTION

As a polymorphic opportunistic human fungal pathogen, *Candida albicans* retains the capacity to broadly adapt to various host and microenvironmental niches. The modulation and management of nutrients, especially carbohydrates, are pivotal for *C. albicans* growth, survival, and environmental flexibility. Aside from serving as substrates to drive aerobic and anaerobic metabolism in yeast, carbohydrates also play fundamental structural roles at the cell wall interface, including the formation of glucans, mannans, chitin, and glycogen (1–4).

Glycogen biosynthesis and catabolism are main metabolic strategies for *C. albicans* and other yeasts to cope with starvation and other severe conditions in the environment. Glycogen metabolism pathways in the model yeast *Saccharomyces cerevisiae* have been well characterized (5, 6). Recently, our laboratory characterized the highly orthologous glycogen metabolism pathways in *C. albicans* (7). In *C. albicans*, glycogen synthesis starts with the glucosyltransferase glycogenin that forms an oligosaccharide core of attached α-1,4-glucose primers. Mature glycogen is then synthesized by elongation of α-1,4 linked chains by glycogen synthase (encoded by *GSY1*), followed with the introduction of branch points (α-1,6 linkages) by the branching enzyme Glc3p, resulting in a mature, dense polymer. Importantly, it was also shown that the capacity to synthesize and catabolize glycogen impacted fitness and virulence of *C. albicans*. Particularly, impaired glycogen synthesis decreased *C. albicans* long-term competitive survival *in vitro* and at the vaginal mucosa in a mouse model of vulvovaginal candidiasis (7).

Several laboratories have investigated and revealed transcriptional control of glycogen synthesis in *S. cerevisiae*, including contributions of the SNF1/PKA pathway and cyclin-dependent protein kinases (*Sc*Pho85p and the cyclins *Sc*Pcl8p and *Sc*Pcl10p) (8–13). However, the key regulatory step of initiating glycogen synthesis is controlled by the reversible dephosphorylation of the synthase *Sc*Gsy2p at its C-terminal Ser650, Ser654, and Thr667 sites (14) by the essential serine/threonine type 1 protein phosphatase catalytic (PPP1C) subunit *Sc*Glc7p (15–20). However, the migration of *Sc*Glc7p to *Sc*Gsy2p is highly dependent on its PPP1 regulatory (PPP1R) subunits. Several of these contain a carbohydrate-binding module family 21 (CBM21) domain, including *Sc*Pig1p (20), *Sc*Pig2p (21), and *Sc*Gac1p; *Sc*Gac1p plays a major role in regulating glycogen synthesis (18, 19). It was previously shown that the *Sc*Glc7p/*Sc*Gac1p holoenzyme dephosphorylates glycogen-bound *Sc*Gsy2p by leveraging the affinity of *Sc*Gac1p for glycogen (15). In support of this, Cheng et al. reported that multiple PPP1R subunits involved in glycogen metabolism across yeast, mammalian, and plant species share a conserved carbohydrate-binding “GVNK” motif (20), likely to drive efficient glycogen synthase activation.

Based on our previous study that indicates glycogen synthesis alters *C. albicans in vivo* fitness (7), along with a lack of studies investigating glycogen synthesis regulatory mechanisms in this human fungal pathogen, we wished to more fully delineate this process. Sequence alignment of *Sc*Gac1p revealed two potential PPP1R orthologs encoded by the *C. albicans* genome, including previously annotated Gac1p and the uncharacterized protein C1_01140p, both of which contained a GVNK motif. Genetic deletion of *C1_01140*, but not *GAC1*, resulted in severely attenuated glycogen synthesis in *C. albicans* that phenocopied the *gsy1*Δ/Δ mutant. Site-directed mutagenesis of the GVNK motif in C1_01140p revealed that it was essential for wild-type levels of glycogen accumulation. Consistent with the *gsy1*Δ/Δ phenotype published previously, the *c1_01140c*Δ/Δ mutant exhibited reduced colonization during murine vulvovaginal candidiasis. Furthermore, fluorescent tagging followed by epifluorescence microscopy and co-immunoprecipitation (Co-IP) assays confirmed that C1_01140p colocalized and associated with *Ca*Gsy1p and *Ca*Glc7p during glycogen synthesis. Therefore, we provide evidence that glycogen synthesis is similarly controlled in *C. albicans* and that C1_01140p is the functional ortholog of *Sc*Gac1p.

## RESULTS

### The uncharacterized gene *C1_01140C*, but not *GAC1*, plays a major role during glycogen synthesis in *C. albicans*

**Fig 1:**
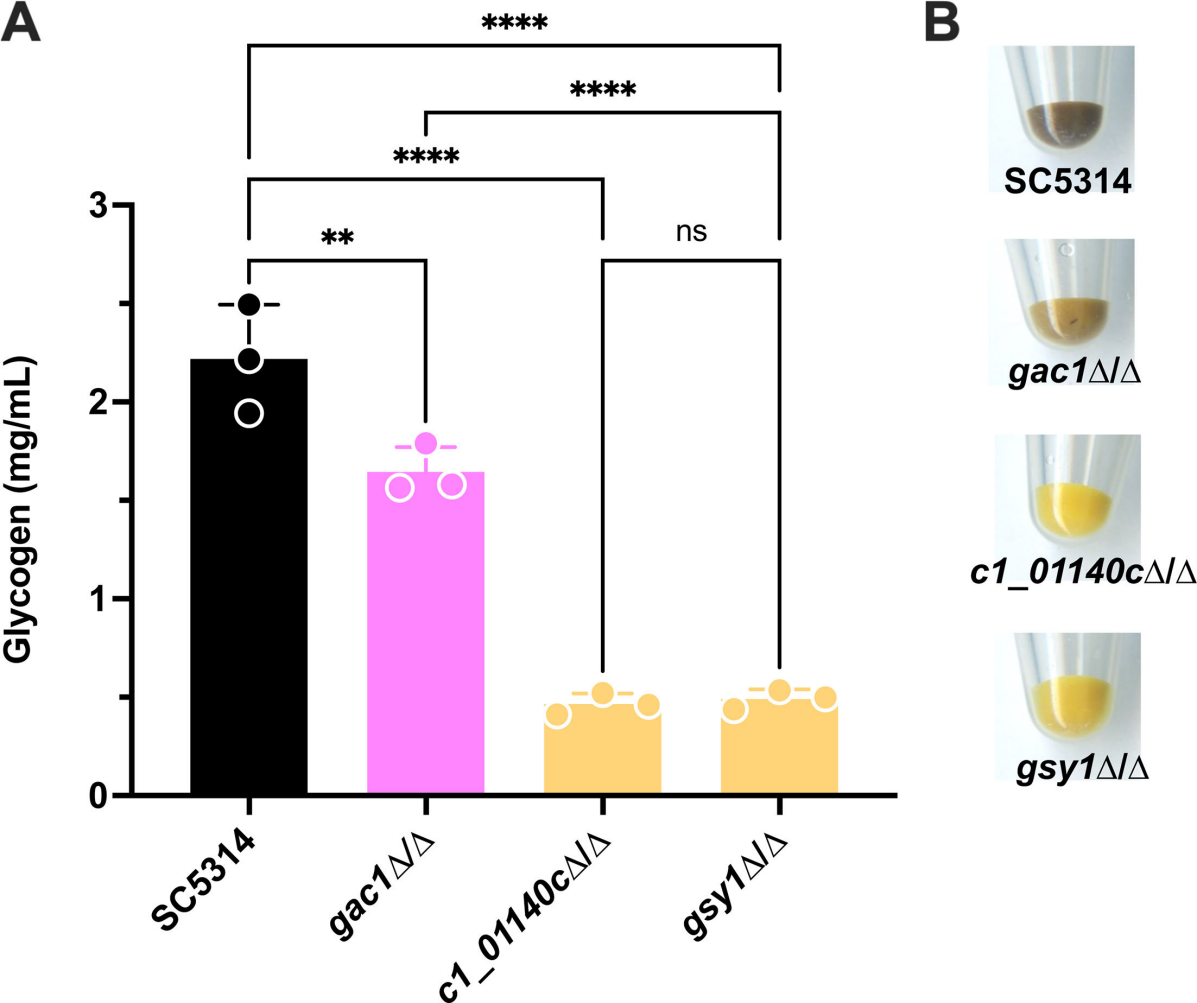
The uncharacterized C1_01140Cp, but not Gac1p, plays a major role in *C. albicans* glycogen synthesis. (**A**) Glycogen concentration of SC5314, *gsy1*∆/∆*, gac1*∆/∆, and *c1_01140*c∆/∆. Glycogen was extracted from 16 h YPD cultures and quantified by iodine staining. The data were interpolated from a standard curve of oyster glycogen and are depicted as the mean + SD. A one-way analysis of variance with Dunnett’s post-test was used for the statistical analyses. **, *P* < 0.01; ***, *P* < 0.001. (**B**) Qualitative iodine staining of the strains mentioned in panel A. Images were captured on a digital scanner and are representative of three independent experiments.

Prior studies in *S. cerevisiae* demonstrated that while multiple PPP1R subunits (including *Sc*Gac1p, *Sc*Pig1p, *Sc*Pig2p, and *Sc*Gip2p) contribute to glycogen synthesis, *Sc*Gac1p was the most impactful (15, 17–21). Sequence alignment of *Sc*Gac1p with the *C. albicans* SC5314 proteome using BLASTp revealed two potential orthologs, currently annotated Gac1p (34.2% identity, systematic name *C7_00660W*) and the uncharacterized protein C1_01140Cp (26.5% identity). Using CRISPR-Cas9-based transformation, we deleted *GAC1*, *C1_01140C*, or *gsy1*Δ/Δ in *C. albicans* strain SC5314. These mutant strains had no discernable growth defect as compared to the wild-type (WT) parental strain under standard laboratory conditions in either yeast nitrogen base (YNB) or yeast peptone dextrose (YPD) medium (Fig. S1). Glycogen synthetic capacity of these strains was evaluated by assessing glycogen accumulation both qualitatively and quantitatively with iodine staining. The *gsy1*Δ/Δ mutant lacking a functional glycogen synthase showed a profound defect in glycogen accumulation as compared to WT, which was consistent with our previous report (Fig. 1A and B) (7). Significant glycogen accumulation defects were observed both in the *c1_01140*c∆/∆ and *gac1*∆/∆ mutants. However, this defect was severe in the *c1_01140*c∆/∆ mutant and phenocopied the *gsy1*Δ/Δ mutant (Fig. 1A and B). These results suggest that C1_01140Cp is the dominant putative PPP1R subunit contributing to glycogen synthesis in *C. albicans*.

### The GVNK motif in uncharacterized C1_01140Cp is required for glycogen accumulation

**Fig 2:**
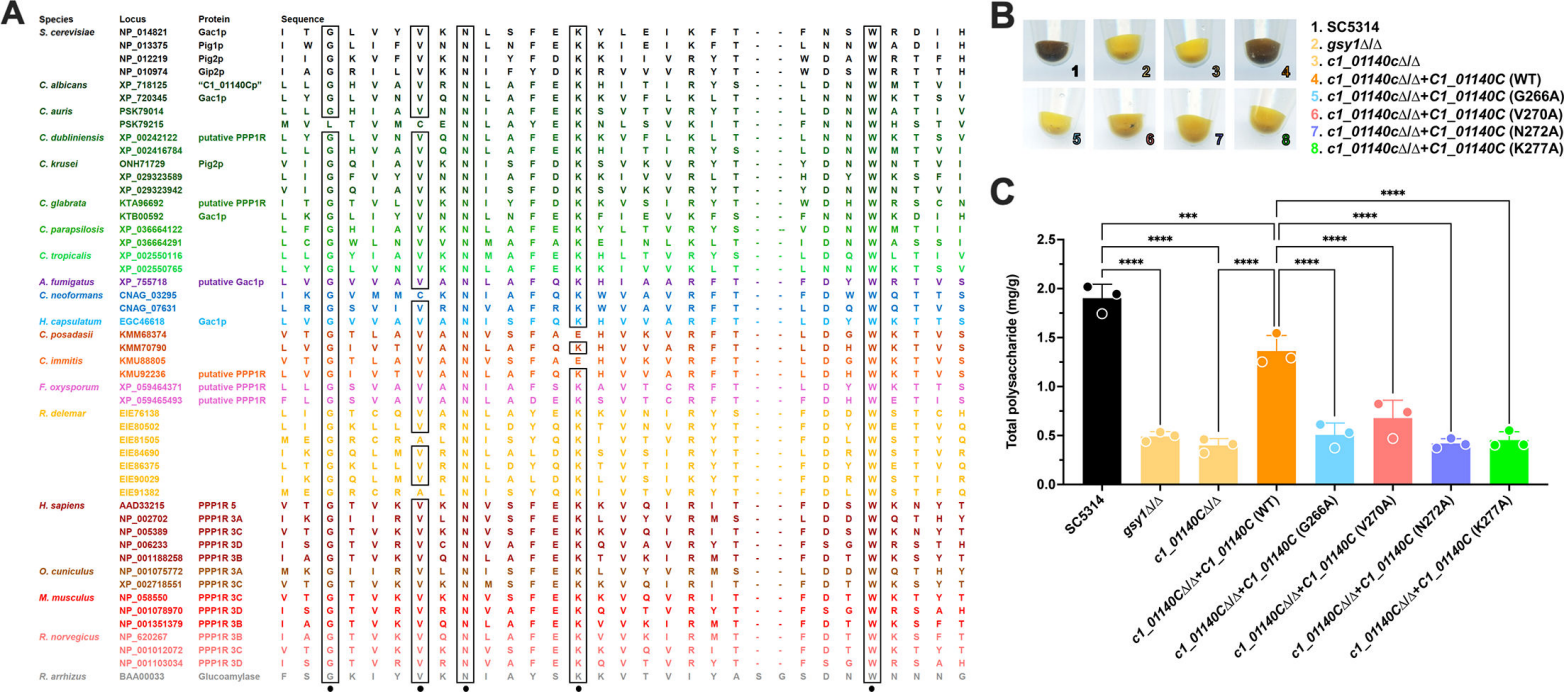
The GVNK motif is highly conserved and required for C1_01140Cp-dependent glycogen synthesis in *C. albicans*. (**A**) A BLASTp alignment depicting conservation of the GVNK motif (black dots) within multiple (putative) PPP1R subunits and glycogen/starch-binding proteins of various species. Black boxes depict additional generally conserved sequences of these proteins. (**B**) Glycogen accumulation phenotypes of strains harboring native *C1_01140C* sequences or those containing point mutations (G266A, V270A, N272A, or K277A) in the GVNK motif as assessed by iodine staining. Images were captured on a digital scanner and are representative of three independent experiments. (**C**) Glycogen content was extracted and quantified by iodine staining and extrapolated using a standard curve of oyster glycogen. The data are depicted as the mean + SD. A one-way analysis of variance with Dunnett’s post-test was used for the statistical analyses. ***, *P* < 0.001; ****, *P* < 0.0001.

Based on protein sequence alignment, C1_01140Cp contains a conserved GVNK motif which is a domain of PPP1R subunits that confers glycogen-binding capacity to multiple CBM family members of diverse species (Fig. 2A) (20). This conservation of the GVNK motif within multiple established or putative PPP1R subunits is observed in mammals (rabbit, mouse, rat, and human), *S. cerevisiae* (e.g., *Sc*Gac1p, *Sc*Pig1p, *Sc*Pig2p, and *Sc*Gip2p), other *Candida* species (*Candida auris, Candida dubliniensis, Candida krusei, Candida glabrata, Candida parapsilosis*, and *Candida tropicalis*), and additional fungal pathogens (*Aspergillus, Cryptococcus, Histoplasma, Coccidioides, Fusarium,* and *Rhizopus*). The GVNK motif is also conserved in some starch degrading enzymes, including the glucoamylase of *Rhizopus arrhizus* (Fig. 2A). To investigate if the conserved GVNK motif in C1_01140Cp is essential for glycogen synthesis in *C. albicans*, a site-directed mutagenesis approach was undertaken to substitute alanine at positions G266, V270, N272, and K277. All transformants were confirmed as having no basal growth differences as compared to the SC5314 parental strain (Fig. S1). Qualitative (Fig. 2B) and quantitative (Fig. 2C) analyses of glycogen accumulation revealed that genetic complementation of *c1_01140*c∆/∆ with native *C1_01140C* restored glycogen synthesis to near WT levels. Locus-specific or gene dosage effects may partly underlie differences observed between WT and native *C1_01140C* revertant strains. However, the revertants of *c1_01140*c∆/∆ complemented with point mutations in each amino acid of the GVNK motif of *C1_01140C* failed to accumulate glycogen and maintained a similar level to that of *gsy1*∆/∆ and *c1_01140*c∆/∆ (Fig. 2B and C). Therefore, the GVNK motif of C1_01140Cp is essential for glycogen accumulation in *C. albicans*.

### The uncharacterized C1_01140Cp associates with PPP1C *Ca*Glc7p and glycogen synthase *Ca*Gsy1p during glycogen synthesis in *C. albicans*

**Fig 3:**
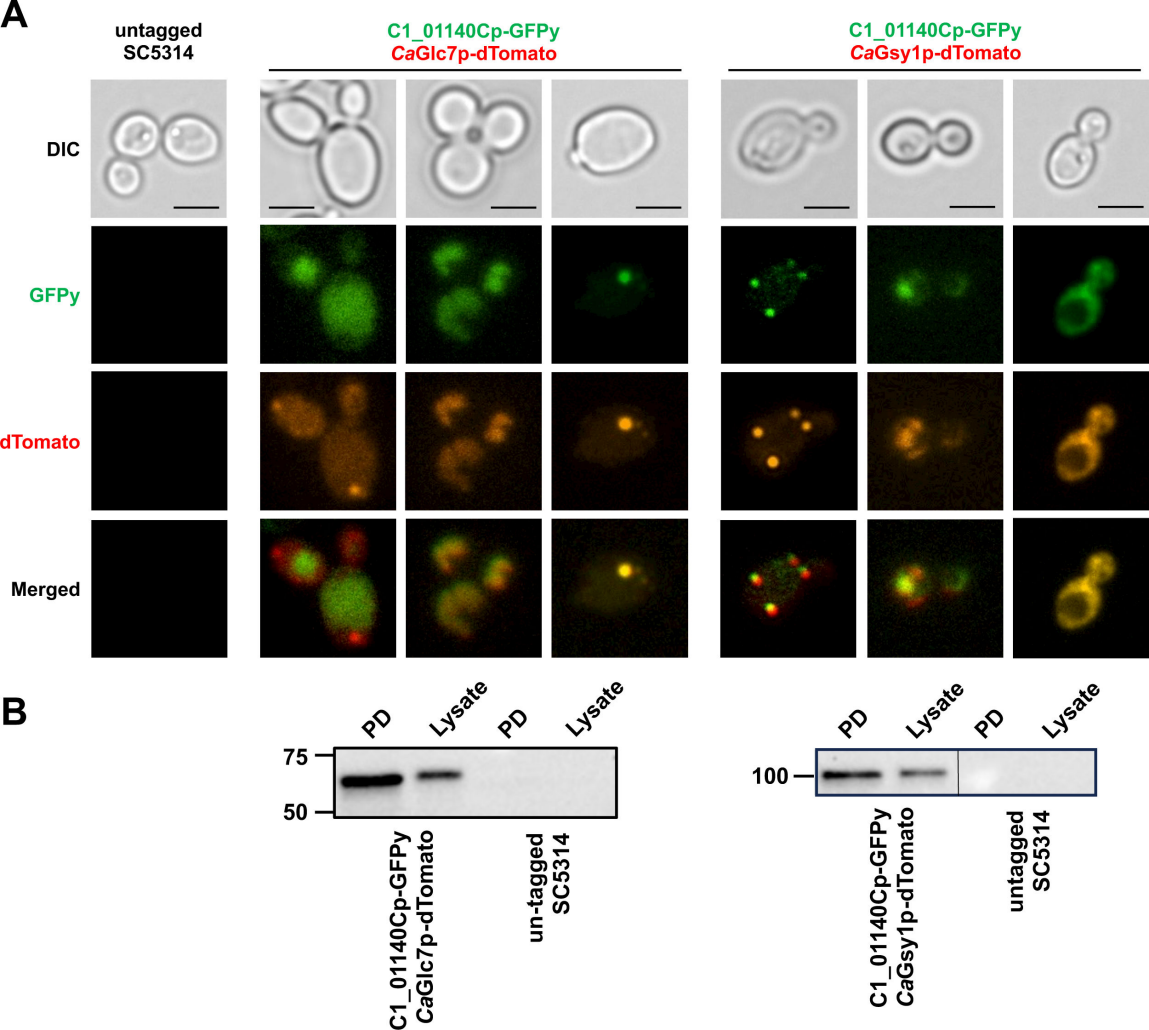
C1_01140Cp colocalizes and associates with the PPP1C subunit Glc7p and glycogen synthase *Ca*Gsy1p in *C. albicans*. (**A**) *Ca*Glc7p or *Ca*Gsy1p were fluorescently tagged with tdTomato and C1_01140Cp was simultaneously tagged with GFPy. Strains were grown in YPD medium for 14 h and epifluorescence microscopy performed using differential image contrast (DIC), fluorescein isothiocyanate (FITC, 488/519 nm), and tetramethylrhodamine (TRITC, 553/590 nm) filters with a 40× objective to determine the cellular localization of the tagged proteins. Digitally captured images are representative of three independent experiments. The scale bar represents 5 µm. (**B**) The bait protein C1_01140Cp-GFPy was immunoprecipitated from the cell lysate using anti-GFP monoclonal antibody conjugated magnetic beads. The prey proteins *Ca*Glc7p-tdTomato or *Ca*Gsy1p-tdTomato in the eluted fraction were visualized by western blot using an anti-tdTomato monoclonal primary antibody and horseradish peroxidase (HRP)-coupled secondary antibody. An untagged SC5314 was used as the negative control. PD: pulled down fraction eluted from anti-GFP magnetic beads. Lysate: cell lysate before Co-IP. Blots are representative of three independent experiments.

Co-immunoprecipitation and yeast two-hybrid system analyses have been previously employed to show that *Sc*Glc7p and *Sc*Gac1p associate during periods of *S. cerevisiae* glycogen synthesis (17). To determine if the potential PPP1R subunit C1_01140Cp is associated with *Ca*Glc7p and/or *Ca*Gsy1p during *C. albicans* glycogen synthesis, we used both epifluorescence microscopy as well as Co-IP approaches with dual fluorescently tagged strains expressing C-terminal fusions of *C1_01140C*-GFPy and *CaGLC7*-tdTomato or *C1_01140C*-GFPy and *CaGSY1*-tdTomato driven by their native promoters. The growth and glycogen accumulation level of these strains were comparable to WT SC5314, indicating that the fluorescent tags did not noticeably impair their functionality (Fig. S1 and S2). Epifluorescence microscopic analysis of *Ca*Glc7p-tdTomato and C1_01140Cp-GFPy revealed consistent cellular colocalization (Fig. 3A), indicating that C1_01140C is likely a PPP1R subunit that interacts with Glc7p. Interestingly, three major localization patterns of glycogen synthase were previously observed in *S. cerevisiae* and categorized as “spot,” “haze,” or “diffuse”, which mirrored those of *Ca*Gsy1p-GFPy localization (Fig. S3) (22). Fluorescence microscopy analysis of *Ca*Gsy1p-tdTomato and C1_01140Cp-GFPy revealed similar colocalization patterns (Fig. 3A). To further explore these interactions, co-immunoprecipitation studies were undertaken. Using the same fluorescently tagged strains, C1_01140Cp-GFPy was used as the bait to potentially pull down *Ca*Glc7p-tdTomato. Co-IP analysis revealed that *Ca*Glc7p-tdTomato (65.4 kDa) was enriched in the co-precipitate indicating that it associated with C1_01140Cp-GFPy during late log-phase growth (Fig. 3B). To investigate whether C1_01140Cp associates with *Ca*Gsy1p, we again conducted a similar Co-IP assay using *Ca*Gsy1p-tdTomato and C1_01140Cp-GFPy-tagged strains, with C1_01140Cp again serving as the bait. Similarly, *Ca*Gsy1p-tdTomato (103.0 kDa) was enriched (Fig. 3B) in the co-immunoprecipitated fraction. Collectively, the fluorescence microscopy and Co-IP results indicate that C1_01140Cp serves as a PPP1R subunit that associates with the PPP1C *Ca*Glc7p and *Ca*Gsy1p during periods of *C. albicans* glycogen synthesis.

### Deletion of C1_01140Cp impacts *C. albicans* fitness during murine vaginitis

**Fig 4:**
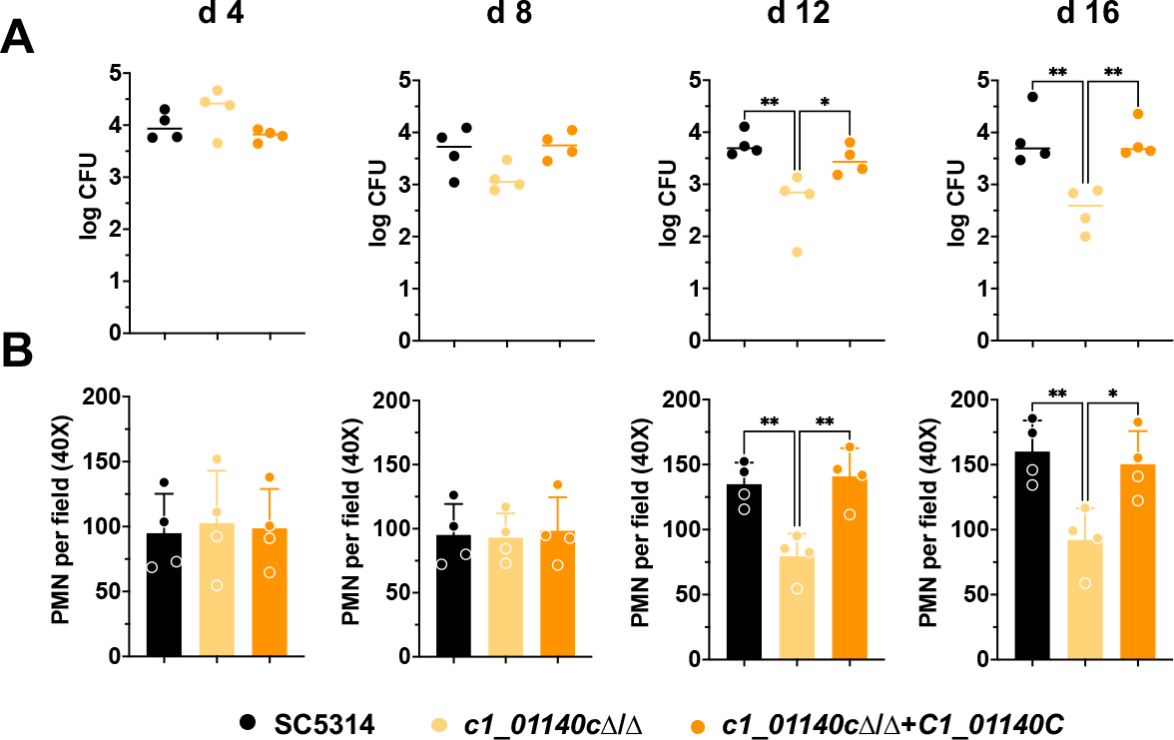
Loss of C1_01140Cp results in reduced *C. albicans* colonization and immunopathology during murine vagintis. Vaginal lavage fluids (VLF) obtained from mice (*n*  =  4 per group) were analyzed at days 4, 8, 12, and 16 post-inoculation for (**A**) fungal burden (medians) and (**B**) PMN recruitment (mean + SD). Significance was determined by using a one-way analysis of variance with the Kruskal-Wallis (colony forming unit (CFU) data) or Tukey’s (PMN data) post-test and denoted as follows: *, *P* < 0.05; **, *P* < 0.01.

Previously, we demonstrated that a *C. albicans gsy1*Δ/Δ mutant exhibited long-term colonization and immunopathological defects at the vaginal mucosa (7). Therefore, we wished to determine if loss of C1_01140Cp would phenocopy the *gsy1*Δ/Δ mutant in the murine model of vulvovaginal candidiasis given its similar glycogen defect (23–25). Fungal burdens were similar among WT, *c1_01140c*Δ/Δ, and revertant strains up to day 8 post-inoculation. However, the *c1_01140c*Δ/Δ mutant displayed significant colonization defects at days 12 and 16 (Fig. 4A). Recruited polymorphonuclear leukocyte (PMN) levels mirrored the fungal burden data, where significant decreases were noted for *c1_01140c*Δ/Δ at days 12 and 16. Collectively, the data demonstrate that genetic deletion of the proposed PPP1R subunit C1_01140Cp impairs long-term fitness of *C. albicans* at the vaginal mucosa.

## DISCUSSION

Intracellular glycogen metabolism is a shared and widely adopted strategy across diverse microbial species that confers fitness advantages at environmental and anatomical site-specific host niches, with the synthesis pathway being one of the key effectors that impacts systemic carbon and energy allocation for such purposes. Multiple studies have demonstrated that genetic disruption of glycogen synthesis leads to a lack of glycogen accumulation in various microbes such as *Lactobacillus acidophilus, Escherichia coli, Streptococcus mutans, Mycobacterium tuberculosis*, and *S. cerevisiae*. This deficiency results in decreased fitness both in laboratory conditions and in model hosts, as specifically observed in the gastrointestinal tract and lungs during murine colonization (16, 26–30). In our previous study, we extended this observation to the human fungal pathogen *C. albicans* and demonstrated that the capacity to synthesize and utilize glycogen positively impacted the long-term fitness in nutrient-deficient environments *in vitro* and on vaginal epithelium *in vivo* (7). Therefore, it is unsurprising that the *c1_01140c*Δ/Δ mutant displayed fitness defects during murine vulvovaginal candidiasis (VVC), given it phenocopies *gsy1*Δ/Δ with respect to impaired glycogen accumulation (Fig. 4). The genetic inputs contributing to glycogen synthesis are multifactorial and complex, as revealed by Zeits and colleagues (31). Using large-scale gene deletion and doxycycline-repressible *C. albicans* libraries and iodine staining, they identified 25 mutants that failed to synthesize glycogen to that of their respective WT control strains. Most of the identified genes remain uncharacterized but others play roles in carbohydrate metabolism (e.g., *PGM1*, *PGK1*, *TPS2*) or regulation of these processes (e.g., *SNF4*). However, among the most attenuated of these mutants was in fact *C1_01140C*, the putative PPP1R subunit investigated here.

**Fig 5:**
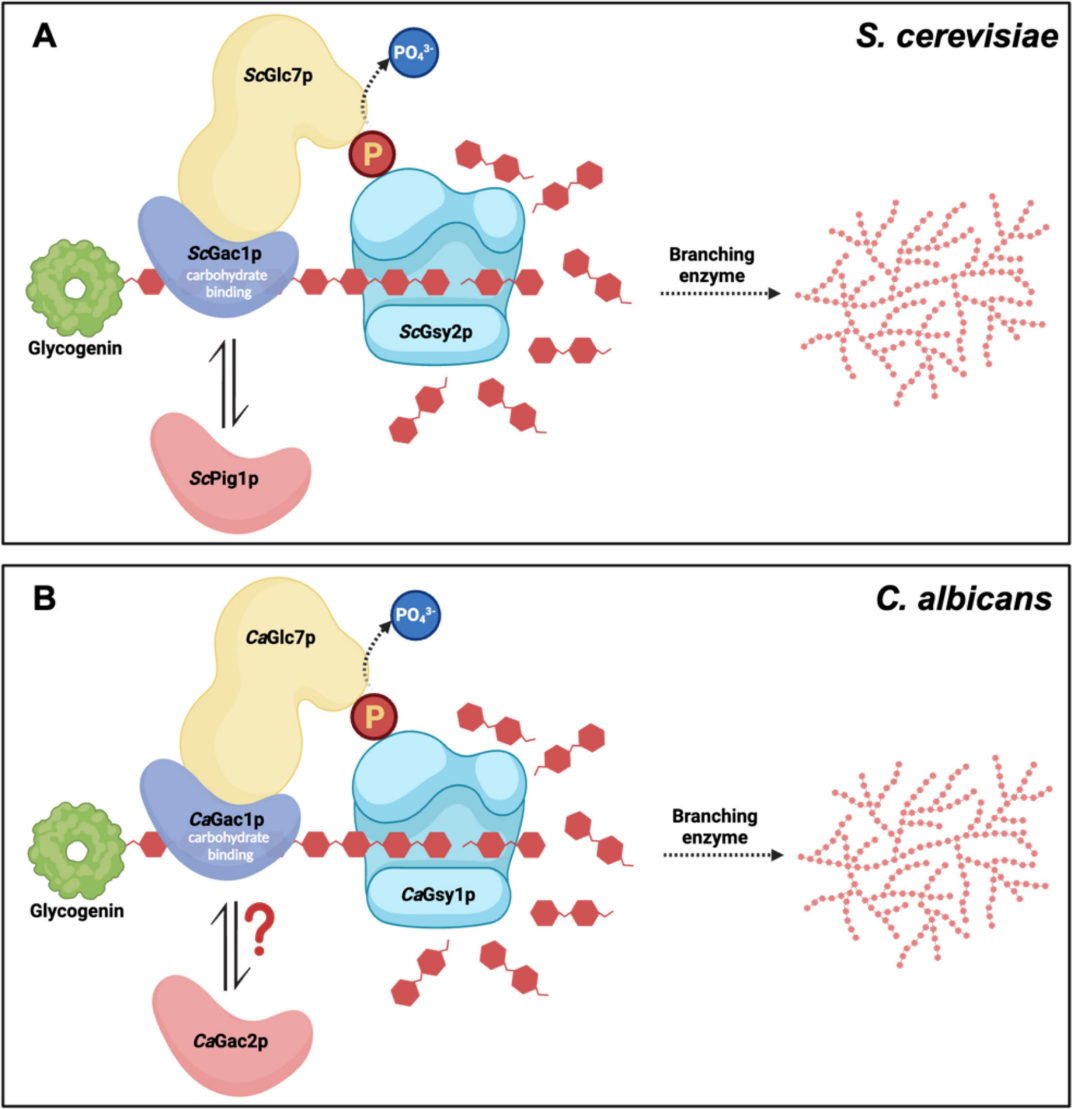
Comparative glycogen synthase activation in *C. albicans* and *S. cerevisiae*. (**A**) In *S. cerevisiae*, the PPP1R subunit *Sc*Gac1p first binds to PPP1C *Sc*Glc7p and targets it to the glycogen synthase *Sc*Gsy2p via its carbohydrate-binding domain. *Sc*Glc7p then dephosphorylates C-terminal residues of *Sc*Gsy2p to initialize glycogen synthesis. *Sc*Pig1p, along with other PPP1R subunits, serves as interchangeable modules to confer diverse localization of *Sc*Glc7p. (**B**) This process is highly orthologous in *C. albicans*, except that newly proposed *Ca*Gac1p (C1_01140Cp) is the major PPP1R subunit that associates with *Ca*Glc7p to activate the lone synthase *Ca*Gsy1p. The newly proposed *Ca*Gac2p (renamed from *Ca*Gac1p) is the only other highly homologous PPP1R subunit found in the *C. albicans* genome and has a limited role in the glycogen synthetic process but may also confer additional yet identified functions. This figure was created using BioRender.

Prior lessons from *S. cerevisiae* reveal that glycogen synthase must first be dephosphorylated by Glc7p for activation and this process is partly mediated by PPP1R subunit-driven localization (5). There are several PPP1R subunits regulating glycogen synthesis that have been characterized in *S. cerevisiae*, including *Sc*Pig1p, *Sc*Pig2p, *Sc*Gip2p, and *Sc*Gac1p. While *Sc*Pig1p was reported to share notable sequence identity (38%) to *Sc*Gac1p, loss of *Sc*Pig1p had only a modest impact on glycogen synthesis compared to the *Sc*Gac1p mutant (20). Unlike *Sc*Pig1p, *Sc*Pig2p shares significant sequence similarity with *Sc*Gip2p, but deletion of *ScPIG2*, *ScGIP2*, or both genes caused no detectable change in glycogen synthesis (20). In *C. albicans*, the only PPP1R subunit that has been annotated currently is Gac1p, without additional *Ca*Pig1p, *Ca*Pig2p, or *Ca*Gip2p orthologs identified. In fact, the only two *C. albicans* proteins that share significant sequence similarity with *Sc*Pig1p were *Ca*Gac1p and uncharacterized C1_01140Cp. Given its annotation, we were surprised to find that the *C. albicans gac1*Δ/Δ displayed only a minor loss of glycogen accumulation as compared to the c*1_01140c*Δ/Δ mutant. These findings were in stark contrast to those described in *S. cerevisiae* by Chang et al. where the *gac1*Δ mutant exhibited a major defect in glycogen accumulation (20). Given the observation above, we propose that uncharacterized C1_01140Cp in *C. albicans* is the true functional ortholog of *Sc*Gac1p and that the currently annotated *C. albicans* Gac1p is likely the functional ortholog of another *S. cerevisiae* glycogen-binding protein. While more detailed experimentation would be required, we hypothesize that this could possibly be *Sc*Pig1p, given its similarly modest impact on *S. cerevisiae* glycogen accumulation (20). Based on our collective data, we propose that *C1_01140C* be named *GAC1* and *C7_00660W* (previously annotated as *GAC1*) be renamed *GAC2* (Fig. 5).

A prior study identified four conserved motifs in *Sc*Gac1p and *Sc*Pig1p and genetic analysis revealed that truncation of “motif I” led to impaired glycogen accumulation in *S. cerevisiae*. (18). Perhaps unsurprisingly, “motif I” encodes the GVNK-containing carbohydrate-binding module. Interestingly, using a sequence homology search of a variety of PPP1R subunits along with other starch- or glycogen-binding proteins from different species, Cheng, et al. reported that the GVNK motif is conserved across diverse PPP1R subunits of rabbit skeletal muscle R_Gl_ and rat liver R_Gl_, *S. cerevisiae* PPP1R subunits *Sc*Gac1p, *Sc*Pig1p, *Sc*Pig2p, and *Sc*Gip2p, human Protein Targeting to Glycogen (PTG) proteins PPP1R3D and PPP1R5, and *Rhizopus* glucoamylase (20). Although the structure and function of the GVNK motif is still not fully delineated, some hypotheses can be raised based on the biological function and physiological processes involving its apparent polysaccharide affinity. The two R_GI_ subunits share domains similar to glycogen-binding motifs of mammalian glycogen phosphorylase, the major glycogen catabolic enzyme (32–35). Two human PTG proteins are members of the human PPP1R family that have been reported to contain glycogen-targeting regions (36). Furthermore, the GVNK motif of *R. arrhizus* glucoamylase is located within loop β23 at site II, which facilitates direct binding to the analogous highly branched plant carbohydrate starch (37). Thus, it is reasonable to conclude that the conserved GVNK motif acts as the starch/glycogen-binding site in PPP1R subunits of *C. albicans* [and likely other human fungal pathogens (Fig. 2A)]. Our site-directed mutagenesis-based alanine scanning of the GVNK motif further supports this hypothesis. As an additional measure of support, yeast PPP1R subunits also contain conserved VXF sequences (38–44) at their N-terminal regions (e.g., *Sc*Gac1p: VRF_73_; *Sc*Pig1p: VRF_57_), which can also be found in *C. albicans* Gac1p (VRF_170_), C1_01140Cp (VHF_132_), and other (putative) PPP1R subunits found in a variety of mammalian and fungal species (Fig. S4). The VXF sequence is a consensus motif for the binding of PPP1R subunits to PPP1C catalytic subunits to drive dephosphorylation of specific cellular targets (43, 45). While this finding has not been extended to yeast, mutational analysis of this sequence would resolve functional conservation with respect to glycogen synthesis.

Based on epifluorescence microscopy, *Ca*Gsy1p, C1_01140Cp, and PPP1C subunit *Ca*Glc7p were generally colocalized with three broadly defined unique localization patterns. A prior study in *S. cerevisiae* described similar localization patterns of GFP-tagged *Sc*Gsy2p that were highly dependent upon the glycogen content in the cell (22). *Sc*Gsy2p was uniformly dispersed throughout the cytoplasm when glycogen was abundant, while it localized to discrete spots within cells under low glycogen conditions. Although not quantified, we more frequently observed the “diffuse” localization patterns of C1_01440Cp, *Ca*Glc7p, and *Ca*Gsy1p at the late log to early stationary phase of growth in our experimental setup, which is the peak period of glycogen synthesis reported in *S. cerevisiae* (8, 16). However, *Ca*Glc7p-tdTomato signal did not always perfectly overlap with C1_01140Cp-GFPy during this growth phase. Aside from glycogen metabolism, *Sc*Glc7p in *S. cerevisiae* regulates diverse physiological processes such as glucose repression, transcription, membrane fusion, sporulation, mitosis, ion homeostasis, and cell wall organization, which are dependent on its subcellular localization (21, 46–48). For example, *Sc*Glc7p is found in diverse subcellular locations at various growth phases, including spindle pole bodies at the start of anaphase until cytokinesis and in the nucleolus throughout the mitotic cell cycle (49). Thus, as a multifunctional phosphatase, *Sc*Glc7p itself has little specificity for substrate catalysis (50). Instead, its specificity is primarily dictated by the regulatory subunits that target *Sc*Glc7p to various enriched cellular substrates associated with cellular senescence, nutrient availability (51), or homeostatic activity (47, 52). For instance, Sds22p is the essential regulatory subunit required for *Sc*Glc7p nuclear localization during mitosis (46, 53). *Sc*Gip1p targets *Sc*Glc7p for meiotic nuclear localization during septum organization and spore wall formation (15, 54). *Sc*Glc7p can also be targeted to the bud neck before cytokinesis and chitin ring assembly (41). Thus, *Ca*Glc7p likely also plays promiscuous cellular roles in *C. albicans*, which may be mediated through assembly with newly named Gac2p or other unidentified alternative substrate binding partners. Using the C-terminal fluorescent protein fusion strains described here, or newly tagged putative PPP1R subunits, time-resolved microscopy experiments under various nutrient or stress conditions could elucidate redundant or unique roles of these binding partners.

Collectively, our data demonstrate that the previously uncharacterized C1_01440Cp (now *Gac1p*) contains a carbohydrate-binding GVNK motif and acts as the major PPP1R-binding subunit that associates with *Ca*Glc7p to form a functional PPP1. This complex is subsequently targeted to the glycogen synthase *Ca*Gsy1p to initiate glycogen synthesis in *C. albicans* (Fig. 5A). These processes are highly orthologous to *Sc*Gac1p-mediated *Sc*Gsy2p regulation in *S. cerevisiae* (Fig. 5B).

## MATERIALS AND METHODS

### Microorganism growth

*C. albicans* strains were maintained as glycerol stocks stored at −80°C. A small amount of stock was spread onto YPD agar and incubated at 30°C for 48 h to obtain isolated colonies. A single colony was transferred to liquid YPD medium and incubated at 30°C with shaking at 200  rpm for 16 h. In some experiments, *C. albicans* was inoculated into YNB medium. Unless specifically noted otherwise, YPD and YNB media routinely contained 2% and 0.5% glucose, respectively.

### Strains, RNAs, and primers

All strains used or generated are listed in Table S1. All primers used for vector and strain construction, as well as CRISPR RNA (crRNA) and guide RNA (gRNA) sequences used in CRISPR-Cas9 gene editing are listed in Table S3.

### Vector construction

All vectors used or generated in this study are listed in Table S2. For alanine scanning of the GVNK motif, the promoter, entire open reading frame (ORF), and terminator of *C1_01140C* was PCR amplified from genomic DNA (gDNA) of SC5314 using primers PrC101140C-XmaI and tC101140C_AmpR-NotI and Platinum SuperFi II PCR Master Mix (ThermoFisher), then cloned into plasmid pDIS3. G266, V270, N272, or K277 amino acids were mutated to alanine using the SuperFi site-directed mutagenesis protocol per manufacturer’s instructions. In short, the whole plasmid sequence was PCR amplified with site mutagenesis primers (e.g., G_A-F and G_A-R and so forth) using Platinum SuperFi II PCR Master Mix (ThermoFisher Scientific). Template plasmid was removed by DpnI (Invitrogen Anza) digestion. All vectors and PCR products were directly transformed into *Escherichia coli* DH5α competent cells, colonies were screened by growth on Luria-Bertani agar plates containing 100 µg/mL ampicillin and subsequent plasmid isolation. All plasmids were verified for the integrity of coding sequence by Nanopore sequencing as fee-for-service (Plasmidsaurus).

### *C. albicans* deletion mutant strain construction

A previously described CRISPR-Cas9-based gene editing approach was used to create mutants, revertants, and protein fluorescent-tagged *C. albicans* strains (55). To generate mutants, gene-specific guide RNAs were designed and disruption repair templates were amplified with primers GOI_CC9KO-F + GOI_CC9KO-R (GOI, gene of interest) using *SAT1*-flipper plasmid (pBSS2) and *CaHygB*-flipper plasmid as templates, which encode resistance to nourseothricin and hygromycin correspondingly. *C. albicans* cells were first treated with transformation buffer and 25 mM dithiothreitol at 30°C and were then washed and mixed with *in vitro* assembled ribonucleoprotein (RNP) complexes composed of target gene-specific 4 µM gRNA, 4 µM universal tracrRNA, 2 µg Cas9 protein, and 1 µg PCR-generated repair templates. Transformation was performed by electroporation at 1.8 kV single pulse. After recovery for 4–6 h at 30°C in YPD medium, cells were plated on YPD plate containing 200 µg/mL nourseothricin (GoldBio) and 600 µg/mL hygromycin B (GoldBio). Selected colonies were then cultured overnight in yeast-peptone (YP) medium containing 2% maltose to induce cassette excision. After selection under lower antibiotic pressure, correct genomic integration and excision of the resistance cassettes were confirmed by growth phenotype on YPD, YPD + 200 µg/mL nourseothricin, and YPD + 600 µg/mL hygromycin plates and PCR amplification using primers GOI_AmpF + GOI_AmpR, FLP_INTF + GOI_AmpR, FLP_INTR + GOI_AmpF, and GOI_DETF + GOI_DETR.

### *C. albicans* revertant and GVNK point mutant strain construction

To generate the complementary strains of *c1_01140*c∆/∆, repair templates were PCR amplified from SfiI-linearized pDIS3-C101140C plasmids (WT, G, V, N, and K) using primers NEUT5L_homology-pDIS-F and NEUT5L_homology-pDIS-R. The revertant strains were constructed using the CRISPR-Cas9 protocol described above except that crNEUT5pDISup crRNA was used during RNP assembly for integration at the neutral *NEUT5* locus. Transformation and genotype confirmation were then performed accordingly as mentioned above with primers NAT1_INTF + NEUT5L_AmpR, PrC101140C_INTR + NEUT5L_AmpF, and C101140C_DETF + C101140C_DETR. All mutants and complementary strains were confirmed by sequencing (Plasmidsaurus) by generating PCR products amplified with NEUT5L_AmpF + NEUT5L_AmpR and SuperFi II high-fidelity polymerase.

### Construction of fluorescently tagged *C. albicans* strains

To generate fluorescent protein-tagged *C. albicans* strains, CRISPR repair templates were generated with overlap extension (OLE) PCR approach as described previously with slight modification. In short, to tag C1_01140Cp with GFPy, five PCR fragments were amplified containing 5´ *NEUT5L* (from linearized pDIS3-tADH1 plasmid), promoter and ORF of *C1_01140C* (from gDNA of SC5314), ORF of GFPy with *ADH1* terminator (from linearized pKE4-GFPy plasmid), *CaHygB* expression cassette (from linearized pHygR plasmid), and 3´ *NEUT5L* (from linearized pDIS3-tADH1 plasmid), using NEUT5L_homology-pDIS-F + N5ADHup-UNIVOL-R, PrC1_01140C_OL-F + C101140C_ORF-linker-R, GFPy-linker-F + tADH1_3´OLE-R, PrHygR-OL-F + tHygR-OL-R, and NEUT5L_OL-F + NEUT5L_homology-pDIS-R, respectively. The same strategy was applied to tdTomato-tagged *Ca*Gsy1p and *Ca*Glc7p, where the NAT1 marker was used instead of *CaHygB* marker for selection. In short, with the example of *Ca*Gsy1p-tdTomato, four PCR fragments were amplified containing 5´ *NEUT5L* (from linearized pDIS3-tADH1 plasmid), promoter and ORF of *GSY1* (from gDNA of SC5314), ORF of tdTomato (from pKE4-tdTomato plasmid), *SAT1* expression cassette with 3´ *NEUT5L* (from linearized pDIS3-tADH1 plasmid), using NEUT5L_homology-pDIS-F + N5ADHup-UNIVOL-R, PrGSY1_OL-F + GSY1_ORF-linker-R, tdTomato-linker-F + tADH1-UNIV-OL-R, and tADH1-UNIV-OL-F + NEUT5L_homology-pDIS-R, respectively. The PCR products were column purified and the concentrations were measured by Nanodrop. The OLE PCR containing SuperFi II polymerase and equimolar amounts of each PCR product from above (100 ng of the longest fragment) was conducted without primer for 15 cycles. Then, a subsequent PCR was performed using the OLE PCR product as a template with primers NEUT5L_homology-pDIS-F + NEUT5L_homology-pDIS-R. The CRISPR-Cas9 transformation was conducted as described above with repair templates of either *CaGSY1*-tdTomato + *C1_01140C*-GFPy or *CaGLC7*-tdTomato + *C1_01140C*-GFPy, and then selected with YPD plus double selection markers. To confirm successful integration of both templates, diagnostic PCRs were performed using primers NAT1_INTF + NEUT5L_AmpR, HygR_DETF + NEUT5L_AmpF, and GOI_DETR + NEUT5L_AmpF. To verify genomic integrity of the tagged strains, PCR products were similarly sequenced after amplification with SuperFi II high-fidelity polymerase and primers NEUT5L_AmpF + tHygR-OL-R and NEUT5L_AmpF + NAT1INTF-FLIP.

### Growth curve

*C. albicans* strains used in this study were grown as described above, washed, diluted to 10^5^ cells/mL in YNB or YPD medium, and 200 µL was transferred to wells of a microtiter plate for the growth curve assay as described previously (7). OD_600nm_ readings were captured at 60-min intervals using a BioTek Synergy spectrophotometer with an incubation temperature of 30°C and orbital shaking at 200 rpm. Experiments were repeated in technical quadruplicate and biological triplicate. Data are expressed as the mean ± standard deviation (SD).

### Glycogen extraction from *C. albicans*

Glycogen isolation was performed as previously described (7). In brief, aliquots of 4 × 10^8^ *C. albicans* cells were harvested and washed twice in sterile water. Wet weight of pellets were recorded, and flash frozen in liquid nitrogen. The pellets were then thawed and resuspended in 20% NaOH and boiled for 1 h with occasional vortexing. Cells were thoroughly resuspended by mixing, chilled on ice, and neutralized with 5N HCl, and centrifuged. Supernatants were transferred to chilled microtubes containing ice-cold 95% ethanol and incubated at −20°C for 30 min. Precipitate was collected by centrifugation and washed twice with 66% ethanol. Pellets were dried at room temperature and redissolved in phosphate buffered saline (PBS), pH 7.0.

### Glycogen iodine staining

A 40× iodine stock solution (20 mg/mL I_2_ in 40 mg/mL potassium iodide (KI)) was prepared and stored protected from light. Cell pellets were harvested from overnight cultures of *C. albicans* grown in YPD and washed twice with PBS. Pellets were then resuspended in 1 mL 1× iodine working solution and incubated at room temperature for 10 min in the dark. Images were acquired using an EPSON digital scanner. Images are representative of three independent repeats. To quantitatively measure cellular glycogen content, extracted glycogen pellets were dissolved in 195 µL PBS then 5 µL iodine stock solution added. Glycogen concentration (mg/mL) was interpolated using a standard curve composed of oyster glycogen as described (7). In short, oyster glycogen was dissolved in dH_2_O, serially diluted, and similarly stained with iodine working solution. The OD_600_ was measured from both experimental and standard samples, blank subtracted values were calculated, and a standard curve was generated by using a linear regression in GraphPad Prism. The concentration was converted to mg/g based on the wet weight of pellets. Experiments were repeated in biological triplicate and values reflect the mean + SD.

### Colocalization by epifluorescence microscopy

Starting with 10^5^ cells/mL, SC5314 WT and fluorescent protein-tagged strains were grown in YPD medium for 14 hours, corresponding to the temporal initiation of glycogen synthesis. Pellets were collected, washed three times, and resuspended with PBS. Wet mounts were prepared on glass slides and observed using a Nikon ECLIPSE Ti2 epifluorescence microscope using differential images contrast, FITC (488/519 nm), and TRITC (553/590 nm) filters with a 40× objective. Images were captured and processed with Nikon NIS-Elements software package (v.5.42.03).

### Co-immunoprecipitation and western blot

Starting with 10^5^ cells/mL, SC5314 WT and fluorescent protein-tagged strains were grown in 20 mL YPD medium for 14 h. Pellets were then collected by centrifugation and washed in PBS three times before resuspended in crosslinking reagent [PBS containing 1 mM dithiobis(succinimidyl propionate), ThermoFisher Scientific] for 30 min at room temperature. The reaction was quenched with 20 mM Tris by adding 500 mM Tris-HCl (pH 7.4) for 15 min. Total protein was isolated as previously described with slight modifications (56). In short, cell pellets were resuspended in PBS supplemented with protease inhibitor cocktail (cOmplete Mini EDTA-free Protease Inhibitor Cocktail, Roche). An equal amount of 0.5 mm acid-washed beads was added to each tube. Cells were mechanically lysed in a Mini-BeadBeater with six rounds of 1-min homogenization at 4°C. After the final round of homogenization, the supernatants were centrifuged at 5,000 rpm for 1 min at 4°C and transferred to new tubes. Supernatants were further centrifuged at 5,000 rpm for 8 min at 4°C, transferred to new tubes, and protein concentrations quantified using the Bradford protein assay (Bio-Rad) following the manufacturer’s protocol. Co-IP assays were performed as described with slight modification (57). Cell lysates containing a total of 2 mg protein was used for Co-IP and incubated with 50 µg TrueMAB Mouse tGFP monoclonal antibody magnetic beads (ThermoFisher Scientific) for 2 h at 4°C on a rocker. The beads were then washed three times with wash buffer (20 mM Tris-HCl, 150 mM NaCl, 0.5 mM EDTA, and 10 mM MgCl_2_). Immunocomplexes were disassociated from magnetic beads with 20 µL Laemmli sample buffer (Bio-Rad) and heated at 95°C for 5 min. The entire eluted fraction and 50 µg cell lysate were separated by SDS-PAGE and transferred to a polyvinylidene fluoride (PVDF) membrane with the Mini-PROTEAN Tetra Vertical Electrophoresis Cell (Bio-Rad). To avoid non-specific recognition of dissociated mouse antibody from the magnetic beads, the tdTomato-tagged proteins were detected with rabbit anti-tdTomato monoclonal antibody (1:1,000 dilution, ThermoFisher), followed by secondary detection with HRP-coupled goat anti-rabbit IgG (H + L) secondary antibody (1:10,000 dilution, ThermoFisher). The blot was incubated in SuperSignal West Pico PLUS Chemiluminescent Substrate (ThermoFisher) for 5 min before imaging with a Gel Doc XR + Gel Documentation System (Bio-Rad).

### Murine model of murine vaginitis

The murine model of VVC was performed as described previously (7, 23–25). In short, groups (*n* = 4) of 6- to 8-week-old female C57BL/6 mice were purchased from Charles River Laboratories and housed in isolator cages mounted on ventilated racks. Mice were administered 0.1 mg β-estradiol 17 valerate (estrogen, E2) dissolved in sesame oil subcutaneously 3 days prior to vaginal lavage or challenge with *C. albicans*. E2 was administered weekly thereafter. Stationary-phase cultures of *C. albicans* strains were washed, counted, and adjusted to 5 × 10^8^ cells/mL in sterile endotoxin-free PBS. Mice were intravaginally inoculated with 10 µL of cell suspension, generating an inoculum size of 5 × 10^6^ blastoconidia. Mice underwent vaginal lavage every 4 days using 100 µL of PBS until day 16 post-inoculation.

### Assessment of vaginal fungal burden and neutrophil recruitment

An aliquot (10 µL) of vaginal lavage fluid (VLF) was smeared onto Tissue Path Superfrost Plus Gold slides (Fisher Scientific), allowed to air dry, fixed with CytoPrep spray fixative (Fisher Scientific), and stored at room temperature. VLFs were centrifuged at 3,600 rpm for 3 min and the supernatants were transferred to new tubes and stored at −80°C. Tubes containing pellets were weighed before and after the removal of the supernatant to calculate the amount of PBS with which to resuspend the pellet for microbiological plating. For assessment of fungal burden, 50 µL aliquots were serially diluted, plated on YPD agar plates containing 50 µg/mL chloramphenicol, and subsequently incubated at 30°C for 48 h. The resulting colonies were enumerated and reported as the median. Slides containing fixed lavage fluids were stained using the Papanicolaou technique to enumerate PMNs as identified by their morphology, staining appearance, and characteristic trilobed nuclei (24, 58). For each smear, PMNs were manually counted in five nonadjacent fields by standard light microscopy using a 40× objective. PMN counts were averaged per field and values were reported as the mean PMN count per group + the standard deviation (SD).

### Figure and graphic construction

Images were constructed in GraphPad Prism (9.3.1), Microsoft PowerPoint (16.67), or BioRender (biorender.com). Any adjustments to brightness or contrast were applied evenly across images. High-resolution, publication-compliant images were rendered with GraphPad Prism (9.3.1) or Adobe Photoshop (23.5.0).

### Statistical analysis

All data were plotted and analyzed for statistical significance using GraphPad Prism (9.3.1). Data were compared using one-way analysis of variance, multiple unpaired *t*-test, and either Dunnett’s or Tukey’s post-tests depending on whether the data were normally distributed as determined by Shapiro-Wilk normality test. Graphs were annotated to indicate significance levels (*, *P* < 0.05; **, *P* < 0.01; ***, *P* < 0.001 and ****, *P* < 0.0001).

## Data Availability

The original contributions presented in the study are included in the article and Supplementary Materials. Further inquiries can be directed to the corresponding author.

## SUPPLEMENTAL MATERIAL

Supplemental material msphere.00575-24-s0001.pdf Supplemental tables and figures.

ASM does not own the copyrights to Supplemental Material that may be linked to, or accessed through, an article. The authors have granted ASM a non-exclusive, world-wide license to publish the Supplemental Material files. Please contact the corresponding author directly for reuse.
