# Supplementary material for: Glycogen synthase activity in Candida albicans is partly controlled by the functional ortholog of Saccharomyces cerevisiae Gac1p
Source: mSphere. 2024 Sep 24;9(10):e00575-24. doi: 10.1128/msphere.00575-24 (PMC11520303; doi:10.1128/msphere.00575-24)
Supplement: Supplemental material — Supplemental tables and figures. [file msphere.00575-24-s0001.pdf]

Species	Strain names used	Parent	Genotype	Reference
C. albicans	SC5314	N/A	Wild type, reference isolate	(1)
C. albicans	*gsy1*Δ/Δ	SC5314	*gsy1*Δ*::FRT+/gsy1*Δ*::FRT+*	this study
C. albicans	*c1_01140c*Δ/Δ	SC5314	*c1_01140c*Δ*::FRT+/c1_01140c*Δ*::FRT+*	this study
C. albicans	*c1_01140c*Δ/Δ+*C1_01140C* (WT)	*c1_01140c*Δ/Δ	*c1_01140c*Δ*::FRT /C1_01140C*Δ*::FRT+ NEUT5L*Δ*::NAT1*-Pr*C1_01140C-C1_01140C-tADH1/NEUT5L*	this study
C. albicans	*c1_01140c*Δ/Δ+*C1_01140C* (G266A)	*c1_01140c*Δ/Δ	*c1_01140c*Δ*::FRT+/C1_01140C*Δ*::FRT+ NEUT5L*Δ*::NAT1*-Pr*C1_01140C-C1_01140C*(G266A)-*tADH1/NEUT5L*	this study
C. albicans	*c1_01140c*Δ/Δ+*C1_01140C* (V270A)	*c1_01140c*Δ/Δ	*c1_01140c*Δ*::FRT+/C1_01140C*Δ*::FRT+ NEUT5L*Δ*::NAT1*-Pr*C1_01140C-C1_01140C*(V270A)-*tADH1/NEUT5L*	this study
C. albicans	*c1_01140c*Δ/Δ+*C1_01140C* (N272A)	*c1_01140c*Δ/Δ	*c1_01140c*Δ*::FRT+/C1_01140C*Δ*::FRT+ NEUT5L*Δ*::NAT1*-Pr*C1_01140C-C1_01140C*(N272A)-*tADH1/NEUT5L*	this study
C. albicans	*c1_01140c*Δ/Δ+*C1_01140C* (K277A)	*c1_01140c*Δ/Δ	*c1_01140c*Δ*::FRT+/C1_01140C*Δ*::FRT+ NEUT5L*Δ*::NAT1*-Pr*C1_01140C-C1_01140C*(K277A)-*tADH1/NEUT5L*	this study
C. albicans	*gac1*Δ/Δ	SC5314	*gac1*Δ*::FRT+/ gac1*Δ*::FRT+*	this study
C. albicans	C1_01140Cp-GFPy+*Ca*Gsy1p-tdTomato	SC5314	*NEUT5L*Δ*::HygR*-Pr*C1_01140C-C1_01140C-GFPy-tADH1/NEUT5L*Δ*::NAT1*-Pr*GSY1-GSY1-dTomato-tADH1*	this study
C. albicans	C1_01140Cp-GFPy+*Ca*Glc7p-tdTomato	SC5314	*NEUT5L*Δ*::HygR*-Pr*C1_01140C-C1_01140C-GFPy-tADH1/NEUT5L*Δ*::NAT1*-Pr*GLC7-GLC7-dTomato-tADH1*	this study
C. albicans	*Ca*Gsy1p-GFPy	SC5314	*NEUT5L*Δ*::NAT1*-Pr*GSY1-GSY1-GFPy-tADH1/NEUT5L*	this study

Table S1. List of *C. albicans* strains used in this study

Vector	Reference
pDIS3	(2)
pDIS3-C101140C (WT)	this study
pDIS3-C101140C (G)	this study
pDIS3-C101140C (V)	this study
pDIS3-C101140C (N)	this study
pDIS3-C101140C (K)	this study
pBSS2	(2)
pHygR	(2)
pDIS3-tADH1	(2)
pKE4-GFPy	(3)
pKE4-tdTomato	(3)

Table S2. List of vectors used in this study

Primer or RNA name	Sequence (5' to 3')
crGSY1_up	CATTCAGTCAGTAAGTCGGT
crGSY1_down	AAGCTTTGATTCGTAAACTC
GSY1_AmpF	CATGACCCAATGTTGGCTTTATTAGT
GSY1_AmpR	AGAAAGGCGTTAAGAACCATTCAGTC
GSY1_CC9KO-F	TTTTTTTCCTTGCATTGTCTCTCGAATTGGCAATTCCTGTGGTTTTCCCAGTCACGACGT
GSY1_CC9KO-R	GTTGCATATTGACTCTGACTTGAGATTGCCTAATTAGTCAGTGTGGAATTGTGAGCGGAT
GSY1_DETF	TACCCCTGATCATGACCTGGAAACC
GSY1_DETR	AGGTTCGTAGTAGGATGGGAACACA
crGAC1_up	TATTATAGACAACTTCCAAC
crGAC1_down	AAGAATAACCTAGAGGATGG
GAC1_AmpF	GTTAAAGTTGGTGGTGGTAGAACAC
GAC1_AmpR	CTCAATACGGAATACCCAACGAGGC
GAC1_CC9KO-F	TCTACACTTATCTTTCACAGTCCTATACAGACACATTTATGGTTTTCCCAGTCACGACGT
GAC1_CC9KO-R	TGATATGATTTCATCTATGACTGTGACAAGGTTCAAGAAGGTGTGGAATTGTGAGCGGAT
GAC1_DETF	CTTCGTCTGGGATTCGAATGGAATTG
GAC1_DETR	CTCAATACGGAATACCCAACGAGGC
crC101140C _up	GTTATGAGGAAAAATGACTA
crC101140C _down	CTCGTATTTGTAACGGCCAA
C101140C _AmpF	TGGTTTGTGTTCATGGATTGTGGCA
C101140C _AmpR	CATGGTTGAAACACAAGTGACAGATGTA
C101140C_CC9KO-F	GGAGTTTTTTTTTCTTCTCTGTTTCTGTACTATGGTCCTTGGTTTTCCCAGTCACGACGT
C101140C_CC9KO-R	CAATCATCCATTATAATACTCAGATTCAAGAGATTCATTATGTGTGGAATTGTGAGCGGAT
C101140C_DETF	GATTCGATGAAGGGCAACGTCAAC
C101140C_DETR	GTTCAGTTGCAGTTGGTTCAGCAG
PrC101140C-XmaI	TCA**CCCGGG**CAAATTGATTGTGGCTTGGTGATGG
tC101140C_AmpR-NotI	TCA**GCGGCCGC**TTGATTGATGTTTCGTCCTGTTTGG
PrC101140C_INTR	GACGGATGGGAATTGTCAAGGTG
G_A-F	TTATTGGCTCATGTTGCTGTTCGAAAT
G_A-R	CAGCAACATGAGCCAATAAGTATTTTC
V_A-F	GTTGCTGCTCGAAATCTTGCTTTTGAA
V_A-R	CAAGATTTCGAGCAGCAACATGACCCA
N_A-F	GCTGCTCGAGCTCTTGCTTTTGAAAAAC
N_A-R	AAAGCAAGAGCTCGAGCAGCAACATGAG
K_A-F	CTTTTGAAACGCATATAACCATTCGTTAC
K_A-R	GGTTATATGCGTTTCAAAAGCAAGAGCT
PrC101140C_OL-F	CCTCGAGGTCGACGGTATCGCGAAACGTAGTCACCAGAAACC
C101140C_ORF-linker-R	GCGGCCGCATCCAGATCTTGTTCCCAAAAACGATGAAATCGTG
tADH1_3'OLE-R	CGAAAACTTGAAACTTGAAAACACCG
PrHygR-OL-F	CGGTGTTTTCAAGTTTCAAGTTTTCGCCATCATAAAATGTCGAGCGTCAAAAC
tHygR-OL-R	CTGCAGAGGACCACCTTTGATT
NEUT5L_OL-F	AATCAAAGGTGGTCCTCTGCAGCAGTGTCGACGTTTAAACGAGCTC
HygR_DETF	GGATGAATTGATGTTGTGGGCTGAAG
PrGLC7_OL-F	CCTCGAGGTCGACGGTATCGGGTGAGAAGTAGCCTCCACGATATTTC
GLC7_ORF-linker-R	GCGGCCGCATCCAGATCTTTTCACAGCCTTCTTTGGTTTTCTTTG
PrGSY1-OL-F	CCTCGAGGTCGACGGTATCGGGCTTCTGCATGGTTGACGAA
GSY1_ORF-linker-R	GCGGCCGCATCCAGATCTAGTTTCTTCTTCACCCAATTCAAAATAACTAATG

tADH1_3'OLE-R	CGAAAACTTGAAACTTGAAAACACCG
GFPy-linker-F	AGATCTGGATGCGGCCGCATGTCTAAAGGTGAAGAATTATTCACTGGTG
dTomato-linker-F	AGATCTGGATGCGGCCGCATGGTTTCAAAAGGTGAGGAGGTG
crNEUT5pDISup	GTAGTAAGACAATATGACTT
FLP-INTF	CGCGCGTAATACGACTCACT
FLP-INTR	CAAGCGCGCAATTAACCCTC
N5ADH1_up-UNIVOL-F	CCTCGAGGTCGACGGTATCG
N5ADH1_up-UNIVOL-R	CGATACCGTCGACCTCGAGG
tADH1-UNIV-OL-F	CGCGTGCATGCTAAGCAAAT
tADH1-UNIV-OL-R	ATTTGCTTAGCATGCACGCG
NAT1_INTF	CCCAGATGCGAAGTTAAGTGCG
NAT1INTF-FLIP	GCATCACCTGGAACAGAAGTTC
NEUT5L_homology-pDIS-F	GCAGATATGAGATAAAAGTTTTAAAGGACAAGAAAAGG
NEUT5L_homology-pDIS-R	ATCTCTAATAATTGCAATTGCAATTGCTTCACATA
NEUT5L_AmpF	GCTGAATCACTTGATAGGATTTAGTTCCATTATGG
NEUT5L_AmpR	GGAATTTCTAGTCACTTGACACGACC

Table S3. List of oligonucleotides used in this study. Engineered restriction enzyme sites are highlighted in bold text and underlined.

SUPPLEMENTARY FIGURES AND LEGENDS

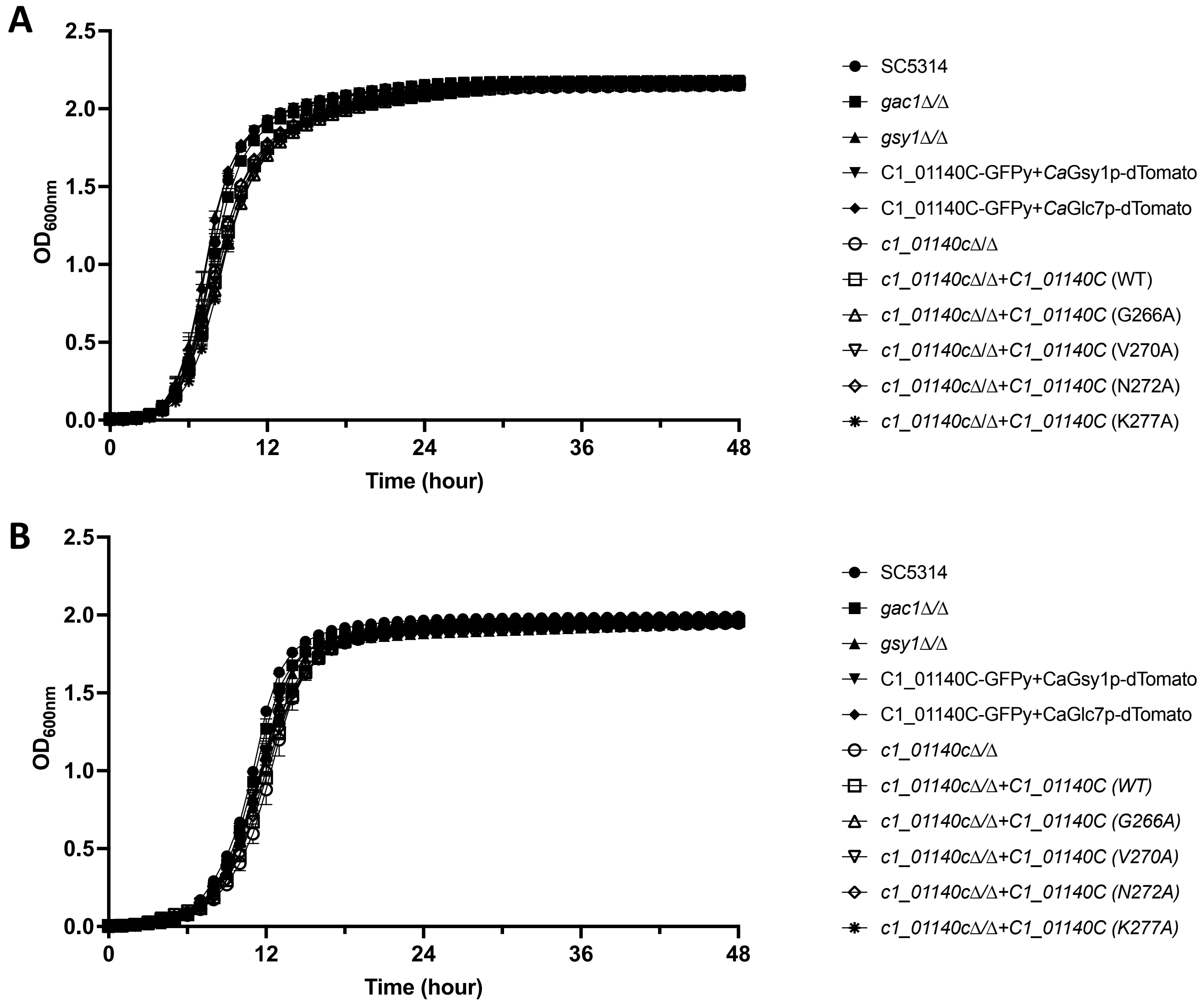

Figure S1. Strains used in this study do not display general growth defect under standard laboratory conditions. WT (SC5314), mutants (*gsy1Δ/Δ*, *gac1Δ/Δ, c1_01140cΔ/Δ*), revertants *c1_01140cΔ/Δ+C1_01140C* (WT) or GVNK point mutants (G266A, V270A, N2272A and K277A), and fluorescence tagged strains (*Ca*Gsy1p-dTomato+C1_01140Cp-GFPy and *Ca*Glc7p-dTomato+C1_01140Cp-GFPy) were inoculated in 96-well plates containing **(A)** YPD medium containing 2% glucose, or **(B)** YNB medium containing 0.5% glucose. Growth was monitored for 48 h at 30°C with double-orbital shaking by measuring OD_{600nm} in a microplate reader. The data is depicted as the mean ± SD. A one-way ANOVA with Dunnett's post-test was used for the statistical analyses.

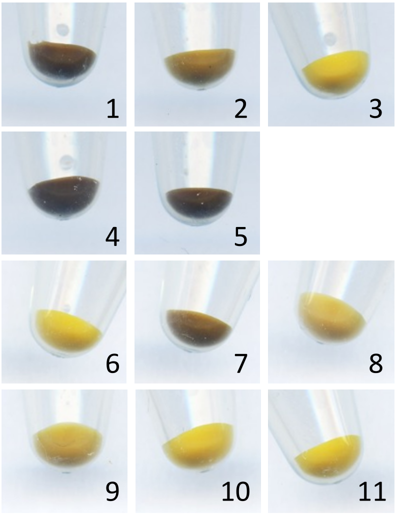

1. SC5314
2. *gac1*Δ/Δ
3. *gsy1*Δ/Δ
4. C1_01140Cp-GFPy+*Ca*Gsy1p-dTomato
5. C1_01140Cp-GFPy+*Ca*Glc7p-dTomato
6. *c1_01140c*Δ/Δ
7. *c1_01140c*Δ/Δ+*C1_01140C* (WT)
8. *c1_01140c*Δ/Δ+*C1_01140C* (G266A)
9. *c1_01140c*Δ/Δ+*C1_01140C* (V270A)
10. *c1_01140c*Δ/Δ+*C1_01140C* (N272A)
11. *c1_01140c*Δ/Δ+*C1_01140C* (K277A)

Figure S2. Qualitative glycogen phenotypes of genetically manipulated strains used in the study. WT (SC5314), mutants (*gac1*Δ/Δ, *gsy1*Δ/Δ, *c1_01140c*Δ/Δ), revertants *c1_01140c*Δ/Δ+*C1_01140C* (WT) or GVNK point mutants (G266A, V270A, N272A and K277A) and fluorescently tagged strains (*Ca*Gsy1p-dTomato+C1_01140Cp-GFPy and *Ca*Glc7p-dTomato+C1_01140Cp-GFPy) were inoculated and grown for 16 h in YPD medium. Cell pellets were washed and stained immediately with iodine solution. Images were captured on a digital scanner and are representative of 3 independent experiments.

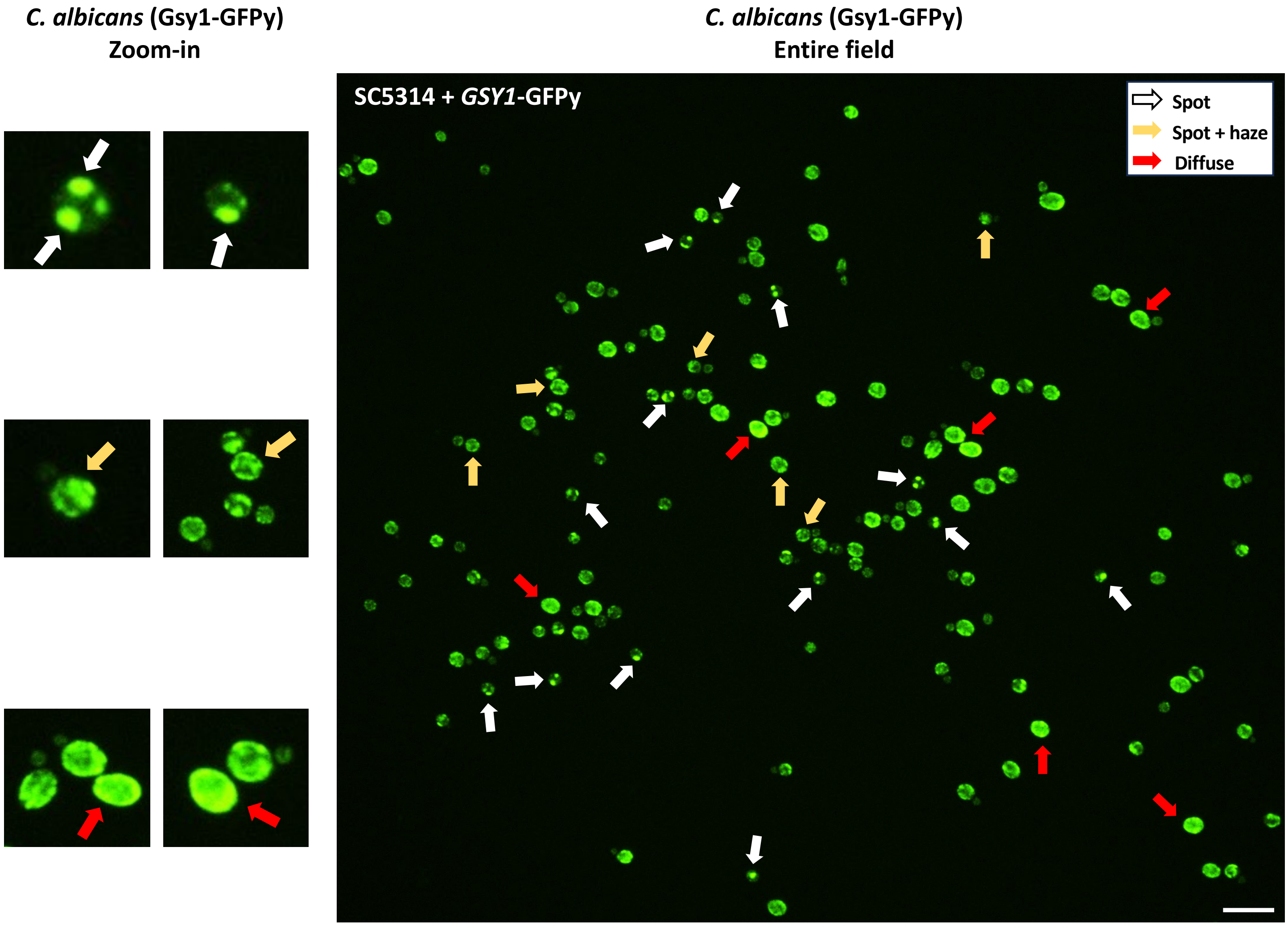

Figure S3. Localization of glycogen synthase in *C. albicans*. SC5314 was engineered to express C-terminally tagged Gsy1p-GFPy. Cells were grown for 16 h in YPD medium and examined by fluorescence microscopy. Three broad localization patterns were observed within the population: bright spots (white arrow), spots accompanied with a haze (yellow arrow), and diffuse cytoplasmic staining (red arrow). The scale bar represents 10 μm. Enlarged images are shown for clarity (left panels).

Species	Locus	Protein	Sequence								
S. cerevisiae	NP_014821	Gac1p	P	E	K	N	V	R	F	A	I
	NP_013375	Pig1p	S	G	K	S	V	R	F	A	A
	NP_012219	Pig2p	R	S	K	S	V	H	F	D	R
	NP_010974	Gip2p	R	S	K	S	V	H	F	D	Q
C. albicans	XP_718125	"C1_01140Cp"	T	Y	K	Q	V	H	F	G	G
	XP_720345	Gac1p	T	R	K	S	V	R	F	A	S
C. auris	PSK79014		S	F	K	S	V	H	F	G	D
	PSK79215		S	P	K	S	V	R	F	A	S
C. dubliniensis	XP_00242122	putative PPP1R	T	R	K	S	V	R	F	A	S
	XP_002416784		T	Y	K	Q	V	H	F	G	G
C. krusei	ONH71729	Pig2p	T	Y	K	Q	V	H	F	G	T
	XP_029323589		N	T	K	S	V	R	F	A	T
	XP_029323942		T	Y	K	Q	V	H	F	G	T
C. glabrata	KTA96692	putative PPP1R	R	S	K	S	V	H	F	D	Q
	KTB00592	Gac1p	S	L	K	N	V	R	F	A	P
C. parapsilosis	XP_036664122		T	F	K	-	V	H	F	G	-
	XP_036664291		P	R	K	S	V	R	F	A	S
C. tropicalis	XP_002550116		T	Y	K	Q	V	H	F	G	G
	XP_002550765		P	R	K	S	V	R	F	A	S
A. fumigatus	XP_755718	putative Gac1p	Y	A	K	A	V	H	F	D	S
C. neoformans	CNAG_03295		C	P	K	Y	V	H	F	D	T
	CNAG_07631		R	S	K	S	V	R	F	A	D
H. capsulatum	EGC46618	Gac1p	F	S	K	A	V	H	F	D	S
C. posadasii	KMM68374		S	T	K	S	V	H	F	E	A
	KMM70790		F	V	K	N	V	H	F	D	A
C. immitis	KMU88805		S	T	K	S	V	H	F	E	A
	KMU92236	putative PPP1R	F	V	K	N	V	H	F	D	A
F. oxysporum	XP_059464371	putative PPP1R	F	S	K	A	V	H	F	D	S
	XP_059465493	putative PPP1R	S	R	K	-	A	D	Y	F	E
R. delemar	EIE76138		P	K	K	S	V	R	F	R	D
	EIE80502		P	T	K	S	V	Q	F	D	K
	EIE81505		K	K	K	S	V	R	F	N	D
	EIE84690		K	K	K	N	V	K	F	D	P
	EIE86375		P	T	K	S	V	Q	F	D	K
	EIE90029		K	K	K	N	V	K	F	D	P
	EIE91382		S	K	K	S	V	R	F	N	D
H. sapiens	AAD33215	PPP1R 5	A	K	K	R	V	V	F	A	D
	NP_002702	PPP1R 3A	G	T	R	R	V	S	F	A	D
	NP_005389	PPP1R 3C	A	K	K	R	V	V	F	A	D
	NP_006233	PPP1R 3D	Q	K	L	R	V	R	F	A	D
	NP_001188258	PPP1R 3B	V	K	K	R	V	S	F	A	D
O. cuniculus	NP_001075772	PPP1R 3A	G	G	R	R	V	S	F	A	D
	XP_002718551	PPP1R 3C	A	K	K	R	V	V	F	A	D
M. musculus	NP_058550	PPP1R 3C	A	K	K	R	V	V	F	A	D
	NP_001078970	PPP1R 3D	R	Q	V	R	V	R	F	A	D
	NP_001351379	PPP1R 3B	V	K	K	R	V	S	F	A	D
R. norvegicus	NP_620267	PPP1R 3B	V	K	K	R	V	S	F	A	D
	NP_001012072	PPP1R 3C	A	K	K	R	V	V	F	A	D
	NP_001103034	PPP1R 3D	R	Q	V	R	V	R	F	A	D

Figure S4. The conserved VXF motif in (putative) PPP1R subunits across multiple species. Alignment depicting the conserved N-terminal VXF motif of (putative) PPP1R subunits across diverse species. Figure was generated using BLASTp and the Clustal Omega multiple sequence alignment tool.

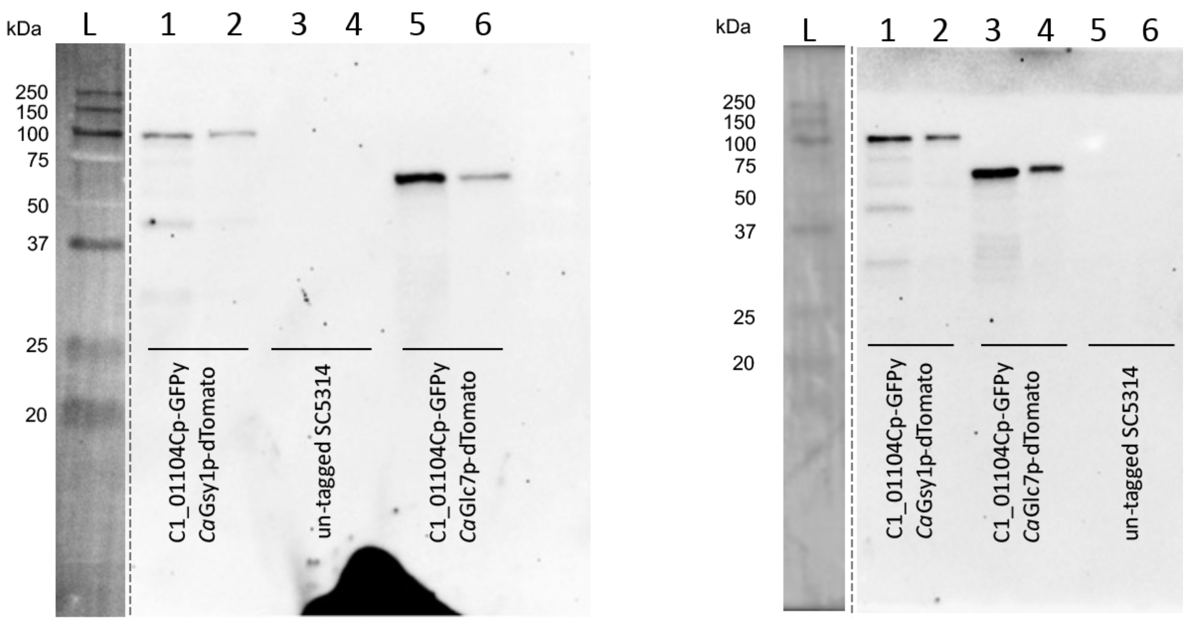

Figure S5. Representative western blot images for Fig 3B. The bait protein C1_01140Cp-GFPy was immunoprecipitated from cell lysates using anti-GFP monoclonal antibody conjugated magnetic beads. The prey proteins *Ca*Glc7p-tdTomato or *Ca*Gsy1p-tdTomato in the eluted fraction were visualized by western blot using an anti-tdTomato monoclonal primary and HRP-coupled secondary antibodies. Entire blot images were obtained using the fluorescence and chemiluminescent channels on the Gel Doc XR+ Gel Documentation System (Bio-Rad) to capture the ladder or HRP-generated signal, respectively. Separation of these images are denoted by the dashed line. Lanes: (L), ladder; (1,3,5), eluate from anti-GFP immunoprecipitation; (2,4,6): cell lysate.

SUPPLEMENTAL REFERENCES

1. Gillum AM, Tsay EY, Kirsch DR. 1984. Isolation of the *Candida albicans* gene for orotidine-5'-phosphate decarboxylase by complementation of *S. cerevisiae ura3* and *E. coli pyrF* mutations. Mol Gen Genet 198:179-82.

2. Liu J, Vogel AK, Miao J, Carnahan JA, Lowes DJ, Rybak JM, Peters BM. 2022. Rapid Hypothesis Testing in *Candida albicans* Clinical Isolates Using a Cloning-Free, Modular, and Recyclable System for CRISPR-Cas9 Mediated Mutant and Revertant Construction. Microbiol Spectr 10:e0263021.

3. Butts A, DeJarnette C, Peters TL, Parker JE, Kerns ME, Eberle KE, Kelly SL, Palmer GE. 2017. Target Abundance-Based Fitness Screening (TAFiS) Facilitates Rapid Identification of Target-Specific and Physiologically Active Chemical Probes. mSphere 2.
